# Persistent Pulmonary Hypertension in Corrected Congenital Left Circumflex Artery to Coronary Sinus Fistula: A Case Report and Literature Review

**DOI:** 10.1155/cric/6985938

**Published:** 2025-12-02

**Authors:** Nihar Jena, Tea Rrapo, Shubham Pahi, Jay Pradhan

**Affiliations:** ^1^Department of Interventional Cardiology, WVU Medicine, Camden Clark Medical Center, Parkersburg, West Virginia, USA; ^2^Oakland University William Beaumont School of Medicine, Auburn Hills, Michigan, USA; ^3^Texas Cardiac Arrhythmia Institute Research, Austin, Texas, USA; ^4^Interventional Cardiology, Oakland University William Beaumont School of Medicine, Auburn Hills, Michigan, USA

**Keywords:** congenital cardiac anomalies, coronary artery fistula, pulmonary hypertension

## Abstract

**Background:**

Coronary artery fistulas (CAFs) are rare congenital anomalies, with the left circumflex artery to coronary sinus (LCx-CS) fistula representing an uncommon subtype. Pulmonary hypertension (PHTN) may arise as a consequence of large, unrepaired CAFs. While most patients experience improvement following surgical repair, there are instances where PHTN persists, necessitating ongoing monitoring and pharmacological intervention.

**Case Description:**

We present the case of a middle-aged woman with no significant medical history who presented with dyspnea. She was found to have PHTN secondary to a large LCx-CS fistula. She underwent surgical ligation of the fistula. Postoperatively, the patient remained symptomatic, with elevated pulmonary arterial pressure persisting, prompting the initiation of triple therapy comprising macitentan, selexipag, and sildenafil. This therapeutic regimen significantly resolved her symptoms and improved her functional capacities.

**Discussion:**

This case highlights the hemodynamic implications associated with a long-standing LCx-CS fistula. Although congenital, such anomalies can remain asymptomatic for extended periods. The most likely hypothesis is that a large chronic fistula can cause irreversible histopathological changes to the pulmonary microvasculature, resulting in a “point of no return” and leading to persistent symptoms even after anatomical correction. In most reported scenarios, the surgical or interventional correction of CAF results in symptom resolution and hemodynamic improvement. However, the described case illustrates an atypical presentation, revealing the potential for sustained elevation of pulmonary arterial pressures. Consequently, this necessitates ongoing pharmacological management.

**Conclusion:**

While LCx-CS fistulas are infrequent and often asymptomatic, chronic fistula cases may result in various complications and symptomatic presentations. The case illustrates a rare case of persistent PHTN in a corrected LCx-CS fistula. Thorough follow-up, early diagnosis, and timely interventions, complemented by pharmacotherapy when necessary, are essential in managing these complex clinical scenarios.

**Summary**

• A CAF should be considered in the differential diagnosis if there is suspicion of a shunt pathology and unexplained PHTN.
• The preferred method for closing a fistula could be percutaneous or surgical, based on anatomy and feasibility.
• PHTN can persist in older patients or late diagnosis from CAF, so pharmacological management along with long-term follow-up is imperative.
• A shared decision-making process with the patient and a multidisciplinary approach are crucial for managing complex congenital cardiovascular disorders.

## 1. Introduction

A coronary artery fistula (CAF) is an anomalous communication between a coronary artery and another blood vessel or cardiac chamber [1]. Most of these fistulas are located in the right coronary artery (RCA), although left-sided fistulas have also been reported. Patients rarely exhibit symptoms; diagnosis is often incidental. Occasionally, symptoms may arise as a result of a chronic left-to-right (L-R) shunt, resulting in pulmonary hypertension (PHTN) [2]. This article details a rare case of a patient who had congenital CAF between the LCx-CS, who developed PHTN; her symptoms persisted despite surgical correction, requiring pharmacological management and long-term follow-up. This case highlights the importance of identifying rare etiologies of PHTN and underscores the role of a multimodal therapeutic approach, combining surgical and pharmacological measures, in managing persistent PHTN.

## 2. History of Presentation

A 51-year-old woman with no past medical history or known birth complications presented as an outpatient with worsening shortness of breath (SOB) and palpitations. During chest auscultation, fine crackles were heard bilaterally; a soft systolic murmur was heard on cardiac auscultation, and trace pedal edema was present. Her laboratory analysis showed mildly elevated B-type natriuretic peptide levels; other parameters were unremarkable. The initial differential diagnoses included coronary artery disease, congestive heart failure (CHF), PHTN, and chronic obstructive pulmonary disease. These were ruled out through clinical assessments and imaging studies, and ultimately, invasive hemodynamics and imaging confirmed secondary PHTN stemming from CAF.

## 3. Diagnostic Assessment

**Figure 1:**
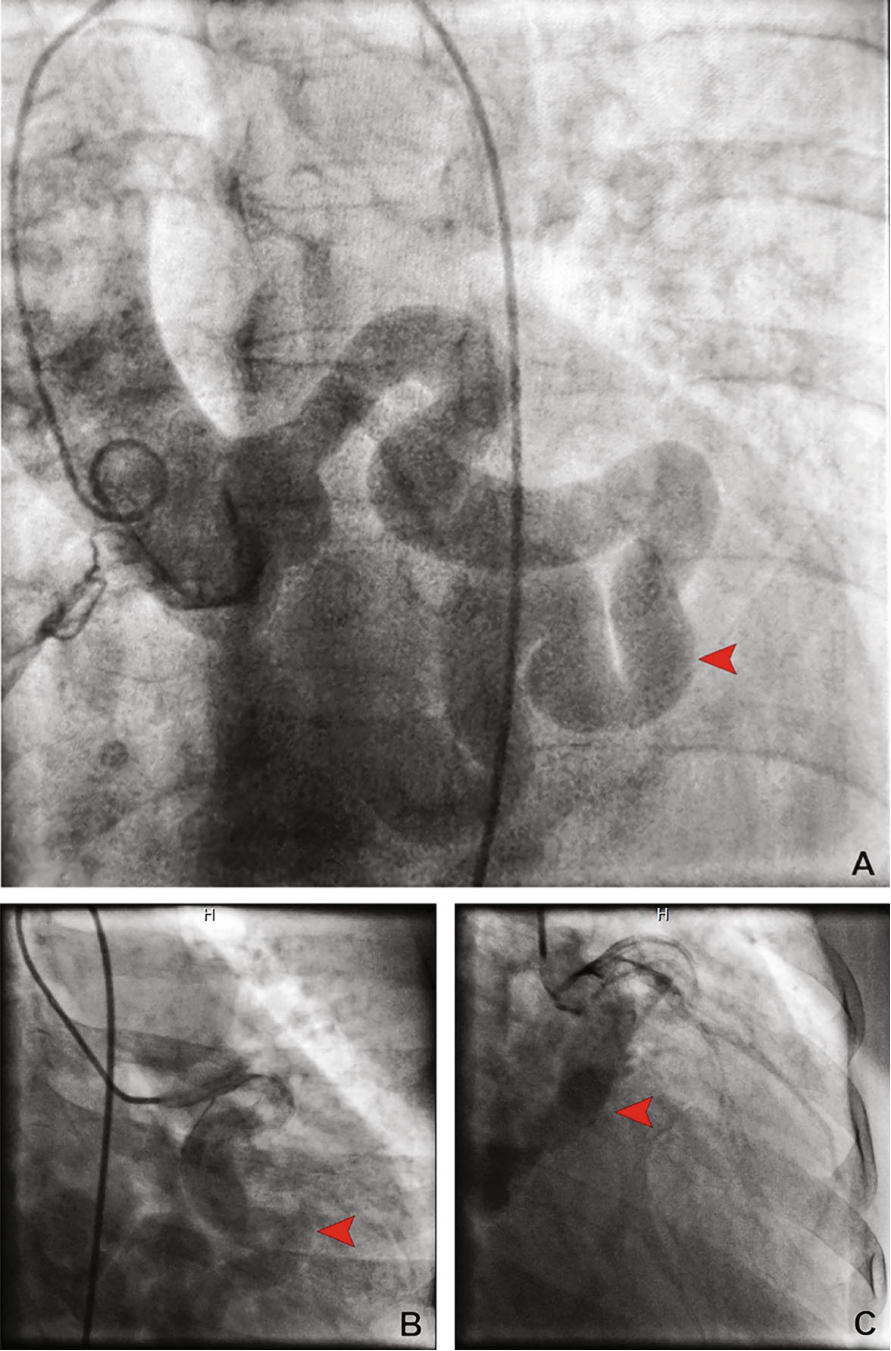
(A) Aortic root angiogram at left oblique caudal (LAO-caudal) view showing ectatic left circumflex artery (red arrow) and fistula to the coronary sinus. (B) Coronary angiogram at LAO-caudal view demonstrating ectatic left circumflex artery (red arrow) and fistula to the coronary sinus. (C) Coronary angiogram at right oblique cranial view showing ectatic left circumflex artery (red arrow) and fistula to the coronary sinus.

**Figure 2:**
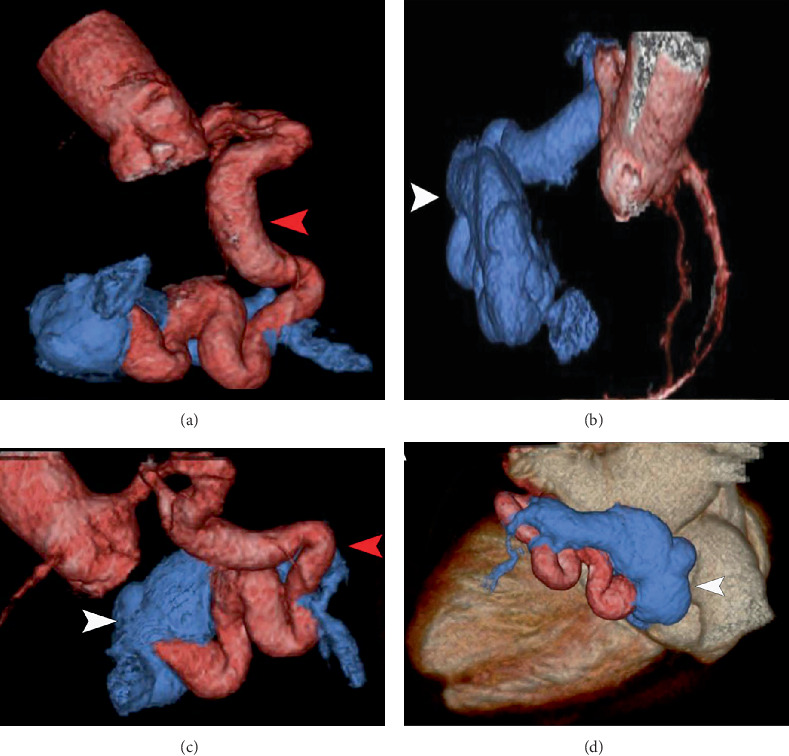
(a–d) 3D reconstruction of coronary CT angiogram showing an ectatic left circumflex artery (red arrow) and fistula to the coronary sinus (white arrow).

The electrocardiogram revealed sinus tachycardia and a right bundle branch block. The echocardiogram showed a preserved left ventricular (LV) ejection fraction and a moderately dilated right ventricle with elevated pressure. A nuclear stress test revealed a reversible perfusion defect in the inferior wall of the LV. A coronary angiogram (CAG) revealed an ectatic tortuous LCx-CS. The left anterior descending artery (LAD) and RCA were normal (Figure 1). The LV end-diastolic pressure was 15 mmHg. The pulmonary capillary wedge pressure was 16 mmHg, and pulmonary artery (PA) pressure was 95/30 mmHg, with a mean of 54 mmHg, and the pulmonary vascular resistance was 7.8 Wood Units. A coronary computed tomography angiogram (CCTA) was performed to better visualize the anatomy, which was consistent with the findings (Figure 2).

## 4. Therapeutic Intervention

Percutaneous coil embolization using 13 and 20-mm coils was unsuccessful due to the large size of the fistula. Surgical CAF ligation was performed. The patient was readmitted due to SOB with elevated troponin levels after a few weeks. CAG revealed a blind LCx pouch with a large thrombus burden, so anticoagulation with warfarin was initiated. Follow-up echocardiography showed a mild decrease in LV function and hypokinesis of the inferolateral wall. Subsequently, the patient experienced atrial flutter and underwent successful ablation.

## 5. Follow-Up

The patient showed persistent symptoms and elevated PA pressure despite CAF ligation. She was followed at the PHTN clinic with serial measurement of PA pressure. Medical management with macitentan, selexipag, and sildenafil was initiated for persistent PHTN. Her symptoms improved in a few months. In a recent follow-up, the patient was asymptomatic and exhibited good exercise tolerance, with a distance of 469.4 m on the 6-min walk test and an oxygen saturation level above 87%. The timeline of the patient management is provided in Table 1.

## 6. Discussion

CAF is an uncommon, incidental finding usually identified during CAG, CCTA, or echocardiography and has a female preponderance. The prevalence of CAF is 0.1%–0.2% of the total CAG, 0.002% of the general population, and 48.7% of all coronary artery anomalies [2]. The site of origin of CAF is in a decreasing trend, with LAD at 42%, RCA at 31%, and LCx at 20% [3, 4]. CS termination occurs in 7% of CAF, though specific data on LCx-CS fistula is not available [5]. In a retrospective study on 6341 patients who underwent CCTA, 0.9% of patients had CAF, of which coronary pulmonary fistula was present in 76.8%, coronary to bronchial artery fistula in 8.9%, coronary to cardiac chamber fistula in 8.9%, combined coronary to pulmonary and coronary to bronchial artery fistula in 3.6%, and coronary artery to superior vena cava fistula in 1.8% [6]. There are a limited number of cases of LCx-CS fistula leading to secondary PHTN that have been reported worldwide.

The etiology may be attributed to congenital or iatrogenic factors. The congenital type usually becomes apparent later in life. Sinusoids play a crucial role in forming the capillary network and facilitating communication. Their regression in the adult heart may result in remnants contributing to the development of fistulas [7]. The iatrogenic form of this condition is increasingly observed due to the growing number of interventions for coronary arteries and electrophysiological procedures. Symptoms are often inversely correlated with the advancement of age [8]. Common presentations include CHF, coronary steal syndrome, PHTN, and arrhythmias. The mechanism of PHTN is similar to other congenital L-R shunt etiologies [2]. Chronic volume and pressure overload culminate in vascular remodeling and progressive elevation of pulmonary vascular resistance. The patient exhibited severe PHTN and elevated PVR despite shunt closure. This finding can be attributed to the late diagnosis of CAF. Traditionally, CAG has been used for diagnosis, but CCTA is now recognized as a noninvasive alternative. Echocardiography, particularly transesophageal echocardiography, can effectively visualize larger fistulas [9].

The treatment of CAF depends on anatomical characteristics, hemodynamic factors, and clinical symptoms. Asymptomatic fistulas with no other defects should be left untreated, while symptomatic cases and severe PHTN require repair through surgical ligation, percutaneous plug, or embolization. Choosing between percutaneous and surgical approaches depends on institutional preference and anatomical intricacy. Both procedures are safe when conducted by skilled operators. In cases of larger fistulas, caution is necessary during percutaneous closure to avoid potential complications. Surgical intervention is recommended for large aneurysmal fistulas, multiple fistulas, and unsuccessful percutaneous procedures [10]. A percutaneous approach was attempted in our case, but the available coil sizes did not anchor due to a substantially large ectatic fistula. A small study of 46 patients revealed that surgical ligation of CAF has a mortality rate of 2%, myocardial infarction in 11% of patients, and a residual fistula in 6% [11]. Surgical ligation has a success rate of 90%–100% when performed with meticulous patient selection. Similarly, percutaneous ligation has a procedural success rate of 81%–100% with proper patient selection and comparable outcomes [2].

Recent case studies discussing the management of LCx-CS fistulas highlight varying patient presentations and treatment outcomes. One report described a 52-year-old woman with a large LCx-CS fistula and mildly elevated PA pressure, who underwent unsuccessful percutaneous closure followed by a successful surgical intervention; postsurgery, she exhibited thrombus formation in the LCx artery pouch as identified through CCTA, yet notably remained asymptomatic with an uneventful recovery [12]. In another instance, an 82-year-old woman with LCx-CS fistula, along with elevated PA pressure, was effectively treated with coronary artery bypass surgery, recovered uneventfully, and remained asymptomatic in follow-up [13]. Additionally, a 58-year-old female patient with LCx-CS fistula and PHTN successfully managed through a percutaneous approach with a vascular plug similarly reported no symptoms during follow-up [14]. It is worth noting that across these cases, patients consistently exhibited the absence of persistent symptoms or significant elevations in PA pressure after percutaneous or surgical repair, underscoring the potential for favorable outcomes in such clinical scenarios.

Despite the surgical ligation of CAF, the patient in our case had persistent PHTN. It is worth noting that despite shunt correction, the prevalence of PHTN in the case of congenital L-R shunt ranges between 2% and 6% in children and 5%–13% in adults [15]. The precise mechanism of persistently elevated PA pressure is not known. The most plausible explanation could be irreversible conformational changes to the pulmonary microvascular. The low prevalence of postcorrection PHTN in children strengthens our hypothesis, as adult patients often present beyond the window, which can be defined as the “point of no return.” PHTN leads to various vascular remodeling of the anatomy, ranging from intimal thickening, medial hypertrophy, luminal dilation, atheromatous changes, thrombotic lesions, and sometimes occlusion [16]. Serial monitoring of symptoms, along with adjustments to pharmacological management, is crucial for achieving a favorable outcome. Additionally, it is worth mentioning multidisciplinary discussion and shared decision-making as integral parts of managing such complex pathologies.

## 7. Conclusion

CAFs are usually benign and discovered incidentally. However, complications such as symptomatic PHTN can arise due to the L-R shunt between LCx and CS. Due to the condition's rarity, managing these complications is challenging, highlighting the need for more data. Individualized management plans, tailored to size, location, symptoms, and associated complications, are crucial for achieving a favorable outcome. Postprocedure follow-up is vital for monitoring persistent PHTN, arrhythmias, and potential ischemia in these patients.

## Figures and Tables

**Table 1:** Timeline of the case.

**Date**	**Events**
Presentation	A middle-aged female presented with shortness of breath and palpitations.
Investigation	Imaging and invasive evaluation revealed a large left circumflex artery to coronary sinus fistula and elevated pulmonary artery pressure.
Procedure	Percutaneous closure was attempted using coil embolization; however, this was unsuccessful due to significant aneurysmal dilation. Eventually, surgical ligation was performed.
Postprocedure	The patient experienced a non-ST elevation myocardial infarction due to thrombosis in the left circumflex artery pouch, which was managed with anticoagulation. Later, she developed atrial flutter, which was treated with ablation.
Follow-up	The patient continued to have persistent pulmonary hypertension and remained symptomatic.
Management	Triple therapy was initiated, consisting of macitentan, selexipag, and sildenafil.
Followed over months	Pulmonary artery pressure decreased over the following months, leading to clinical improvement. The patient's 6-min walk distance exceeded 460 m, and her oxygen saturation (SpO2) was greater than 87%.

## Data Availability

Data sharing does not apply to this article as no new data were created or analyzed in this study.

## Nomenclature

CAF: coronary artery fistula
CAG: coronary angiogram
CCTA: coronary CT angiogram
CS: coronary sinus
LCx: left circumflex artery
PHTN: pulmonary hypertension
